# Oligotrophic bacteria and pathotrophic fungi moderate multitrophic interactions in semi-arid and arid environments

**DOI:** 10.1186/s40793-025-00788-1

**Published:** 2025-11-19

**Authors:** Premchand Maisnam, Thomas C. Jeffries, Jerzy Szejgis, Dylan Bristol, Uffe N. Nielsen

**Affiliations:** 1 Hawkesbury Institute of Environment, Western Sydney University, Penrith, NSW Australia; 2 W.K. Kellogg Biological Station, Michigan State University, Hickory Corners, MI USA; 3 School of Science, Western Sydney University, Penrith, NSW Australia; 4 Department of Forest Ecology and Management, Swedish University of Agricultural Sciences, Umeå, Sweden

## Abstract

**Background:**

Future climate projections indicate shifts in intra-annual precipitation patterns, with intensified rainfall events and prolonged dry periods. These changes may alter soil biotic communities and their interactions within food webs, particularly in semi-arid and arid ecosystems. However, the influence of varying rainfall regimes and increasing aridity on multitrophic associations in drylands remains poorly understood.

**Methods:**

We leveraged a long-term rainfall manipulation experiment across 6 dryland sites in eastern Australia, including 2 arid ecosystems and 4 semi-arid ecosystems with different levels of rainfall (coefficient of variation, CV), resulting in 3 different climatic conditions. Surface soil was collected from replicated plots subjected to increased (+ 65%) or reduced (− 65%) rainfall relative to ambient conditions using rainout shelter. We characterized bacteria, fungi, protist, and nematode communities using high-throughput amplicon sequencing targeting 16S rRNA, ITS, 18S rRNA and 28S rRNA regions, respectively. Multitrophic co-occurrence network among these groups were constructed to assess biotic responses to rainfall and climatic variations.

**Results:**

Soil biotic community composition in drylands was primarily shaped by environmental conditions, with rainfall treatments exerted no main effect. Belowground multitrophic co-occurrence networks varied significantly across climatic conditions, with aridity promoting positive bacterial associations. Bacteria, fungi, protist formed highly connected modules, and their interactions were central in maintaining multitrophic network structure. Oligotrophic bacteria and pathotrophic fungi emerged as dominant keystone taxa, with their abundance strongly influenced by mean annual precipitation (MAP), underscoring the role of long-term climatic gradients over short-term rainfall changes.

**Conclusions:**

Our findings demonstrate that increasing aridity and rainfall variability reshape soil multitrophic networks in drylands, favoring communities dominated by stress-adapted taxa. The concurrent rise of fungal pathotrophs, potentially driven by declines in protist consumers, may undermine ecosystem resilience. Incorporating multitrophic perspectives into climate impact assessments is essential for anticipating and mitigating emerging threats, such as rising soil-borne pathogens in dryland ecosystems.

**Supplementary Information:**

The online version contains supplementary material available at 10.1186/s40793-025-00788-1.

## Introduction

Global warming is expected to amplify the hydrological cycle, leading to more extreme variation in intra-annual precipitation, resulting in fewer but larger rainfall events and longer periods of dryness [1]. Such changes pose a significant threat to life on Earth, especially in regions that depend predominantly on rainfall to support ecosystem function, including arid and semi-arid areas [2]. Soil biota play a crucial role in dryland ecological processes, such as nutrient cycling and net ecosystem productivity [3, 4]. There is substantial evidence that these organisms are highly sensitive to changes in rainfall patterns and increasing aridity, which can significantly impact the structure and composition of soil biological communities [5, 6]. While bacterial and fungal co-occurrence have been extensively examined under climate change in semi-arid and arid ecosystems, the co-occurrence patterns across multitrophic groups including bacteria, fungi, protists and nematodes that occupy distinct trophic levels (e.g., primary producers, decomposers, consumers) remain relatively under-investigated [7].

Belowground communities are complex and diverse, encompassing a broad array of organisms, forming intricate food webs or ecological networks that regulate ecosystem processes such as carbon and nutrient cycling [8–10]. Bacteria and fungi constitute the base of the soil food web, acting as the primary agents of most soil functions, while protists and nematodes play important roles as biological regulators of prey bacteria and fungi [10, 11]. Protists and nematodes include multiple trophic groups, including herbivores, omnivores, predators, and microbial feeders, and thereby influencing microbial communities and directly or indirectly, affecting plant health and biotic soil processes [10–12]. Alterations in rainfall and soil moisture can significantly affect these dynamics. For instance, reduced precipitation can alter nematode and protist abundance, particularly bacterivores, with cascading effects on microbial communities and nutrient cycling [13–16]. Furthermore, these taxa exhibit diverse life histories, including differences in reproduction, life cycles, adaptability, and feeding habits, can respond to environmental disturbances and climate change in markedly different ways [15, 17]. Therefore, research that investigates multitrophic dynamics under changing environmental conditions is essential for anticipating ecological consequences in drylands and beyond.

Ecosystem functioning depends on complex interactions among biological, physical and chemical soil components [18, 19]. Within these interactions, phylotypes in soil food webs that share similar environmental and resource preferences may group together to form dense ecological clusters or modules, potentially influencing ecosystem functions [8, 20]. Network analysis provides a useful framework to study these dynamics. It can reveal how microbial and multitrophic associations change across environmental gradients or disturbances, and help identify keystone groups that disproportionately contribute to network stability [21, 22]. Previous studies have shown evidence of strong microbial co-occurrence forming well-defined modules across climatic gradients, and increasing aridity may alter the relative abundance of these modules through reduced water availability and changes in soil properties [8, 23]. Identifying key biotic and abiotic drivers along climatic gradients is therefore a global priority to predict how soil communities will respond to changing environmental conditions.

This study investigates how variations in rainfall and aridity influence soil multitrophic interactions in semi-arid and arid ecosystems. Previous studies, including our earlier work at these sites [24], have shown that arid conditions favor stress-adapted taxa that dominate microbial community structure [25, 26]. Building on this evidence, we ask how such dominance shapes interactions across multiple trophic levels. To address this gap, we propose two main hypotheses. Hypothesis 1 posits that long-term climatic conditions, reflected by site-level mean annual precipitation (MAP), shape protist and nematode communities similarly to microbial communities. Increasing aridity is expected to enhance compartmentalization within multitrophic co-occurrence networks, leading to tightly clustered modules with fewer cross-trophic links. These structural shifts may alter trophic interactions, nutrient and energy flows, and overall ecosystem resilience under water-limited conditions. Hypothesis 2 proposes that stress-adapted taxa, capable of persisting under low nutrients or drought, occupy central positions within these networks, mediating multitrophic interactions and contributing to belowground community stability under stress. Together, these hypotheses aim to improve understanding of soil food-web responses to rainfall variability and climatic conditions, offering insights into the health and stability of semi-arid and arid ecosystems under climate change.

## Material and methods

### Experimental design and sampling

**Fig. 1:**
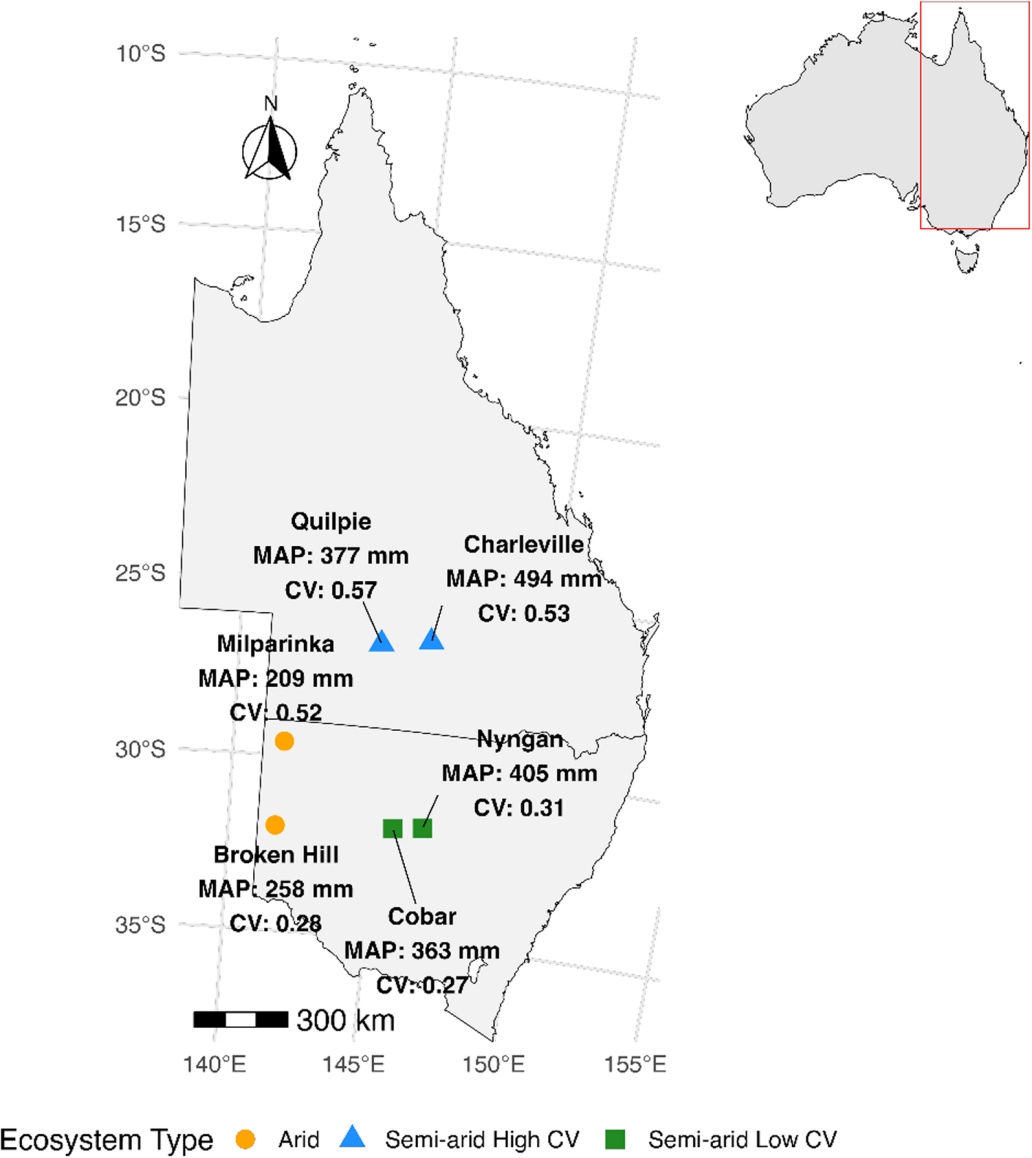
Map of 6 study sites across New South Wales (NSW) and Queensland (QLD), Australia, classified as arid (Broken Hill, Milparinka, NSW), semi-arid low CV (Cobar, Nyngan, NSW), and semi-arid high CV (Charleville, Quilpie, QLD). Mean annual precipitation (MAP) and coefficient of variation (CV) of rainfall are shown next to each site. Background outlines show NSW and QLD boundaries, and an inset map of Australia (top right) highlighting the study region. Map generated in R using the rnaturalearth package; coordinates projected to Australian Albers Equal Area (EPSG:3577)

This study leveraged long-term rainfall manipulation facilities at six sites in western New South Wales (NSW) and south-western Queensland (QLD), established to assess the effects of altered rainfall regimes on ecosystem structure and function in Australian semi-arid and arid ecosystems. The sites have different vegetation and soil properties, with all environmental values calculated for the year 2020 (Fig. 1, Table S1). Across all sites, mean annual precipitation (MAP) ranged from 227 to 462 mm, with an interquartile range (IQR) of 144 mm (260–404 mm). Similarly, mean annual temperature (MAT) showed an IQR of 2.3ºC (19.2–21.5 °C), and aridity index (AI; calculated as the ratio of mean annual precipitation to potential evapotranspiration [27]) had an IQR of 0.12 (0.16–0.28) (Fig. 1, Table S1). Long-term MAP and MAT values were derived from WorldClim version 2.1 (1970–2000), which provides spatially interpolated monthly climate data at a high spatial resolution (~ 1 km) based on ground station records and satellite data, including MODIS [28]. The 6 sites span distinct rainfall regimes, with rainfall increasing from west to east and greater interannual variability (CV; coefficient of variation, calculated as standard deviation divided by mean precipitation [1]) observed at the northern sites (CV ranging from 0.28 to 0.57; IQR of 0.25 (0.28–0.53). For this study, semi-arid sites with higher CV were grouped as ‘Semi-arid High CV’, whereas those with lower CV were groups as ‘Semi-arid Low CV’, and the 2 remaining arid sites were collectively grouped as ”Arid’, making three different climatic conditions. By creating these groupings, studies can systematically compare how changes in rainfall variability (high vs. low CV) and aridity (semi-arid vs. arid) influence biotic assemblages. This helps identify patterns and drivers of ecological change across climatic conditions.

The sites were established in 2016 with 3 different rainfall treatments, representing ambient (control), reduced (− 65%) and increased (~ 65%, but lower given inefficiencies in the transfer of water) rainfall, with 3 replicates at each site. The rainout shelters used a 3 × 3 m plot design, combining rainfall exclusion with collection and gravity induced transfer of water to create the treatments. Reduced rainfall treatment plots had corrugated plastic roof slats that remove approximately 65% of the incoming rain. The excess rainfall from reduced rainfall plots were collected via gutters and transferred via polyethylene piping to adjacent increased rainfall plots. The shelters above the ambient and increased rainfall plots had a bird mesh to simulate the shelter effect. The study sites are all registered under the international collaborative program Drought Network (Drought Net, https://wp.natsci.colostate.edu/droughtnet/), and the rainfall reduction treatment simulates a 1-in-100-year drought. Further details on site characteristics and experimental design are provided in previous publications [24, 29].

Prior to collecting soil samples, we conducted vegetation surveys of each plot, focused on the core 1.5 × 1.5 m quadrat to avoid edge effects, counting the number of individuals of each plant species in each plot to assess composition and species richness. Standing biomass (g m^−2^) was then estimated using allometric relationships based on the percentage cover of individual plant species via relationships for the same species assessed at individual sites [30]. When we were unable to determine the specific species, we used allometric relationships for the most similar functional type.

Eight soil cores (3.5 cm diameter, 0–10 cm deep) were collected randomly within the central 1.5 × 1.5 m quadrat and combined to form a composite sample for each plot, with 3 replicate plots of each treatment per site resulting in a total of 9 samples per site. Samples were transported to Hawkesbury Institute of Environment (HIE), Western Sydney University (WSU), subsampled for chemical analyses and DNA extraction with soils for the latter stored at − 20 °C until further analyses.

### Soil properties

Soil moisture content was estimated by oven drying ~ 10 g fresh soil at 105 °C for 42 h. Soil pH was measured in a 1:5 soil:water slurry using a calibrated pH meter (S20 SevenEasy Mettler Toledo). Approximately 5 g air dry soil were mixed with 25 mL distilled water in a centrifuge tube and shaken for 1 h before measurements were taken. Total soil carbon (TC) and total nitrogen (TN) contents were determined after finely grinding the soil to a powder, using a CN analyzer (TruMac, LECO Corporation, St. Joseph, MI, USA) based on the Dumas combustion method (LECO Corporation, 2003) with a combustion temperature of 1350 °C. Total P was determined on pellets made of 1 g air-dried soil mixed with 2.5 g boric acid powder using an Epsilon 4 Benchtop X-ray fluorescence (XRF) spectrometer (Malvern Panalytical, Malvern, UK).

### Amplicon sequencing and processing

Bacterial, fungal and protist DNA was extracted from ~ 0.5 g of soil after thawing at room temperature using the DNeasy PowerSoil DNA Isolation Kit (QIAGEN, Germany), following the manufacturer’s protocol. For nematode samples, 100 g of homogenized soil was processed using a modified Baermann funnel technique over 3 days, as described by Wang et al. [31]. In this method, soil was placed on a double-layered tissue paper supported by a mesh in a funnel filled with water, allowing active nematodes to migrate out of the soil and into the water over 72 h. The eluate was collected and concentrated by allowing nematodes to settle for 30 min at room temperature, followed by centrifugation at 500 × g for 5 min. A 500 µl of the concentrated suspension solution was subsequently used for DNA extraction using the DNeasy PowerSoil DNA Isolation Kit. This approach enables direct extraction of nematode DNA from a larger soil sample, thus improving the robustness of the nematode community data, but induce some bias due to differences in extraction efficiency of different nematode species including those in anhydrobiotic states. The concentration and purity of all DNA extracts were assessed using a Qubit 4 Fluorometer (Thermo Fisher Scientific, Waltham, MA, USA) and NanoDrop spectrophotometer (Thermo Fisher Scientific), respectively, adhering to standard protocols [32, 33]. DNA samples were then submitted to the Western Sydney University Next-Generation Sequencing Facility for amplicon library preparation and sequencing.

Amplicon libraries were prepared using the NEBNext Ultra II Q5 Master Mix (New England Biolabs, Ipswich, MA, USA) in accordance with the manufacturer’s instructions. Specific primer pairs were employed to target different taxonomic groups. For bacteria, the 341F (5′-CCTACGGGNGGCWGCAG-3′) and 805R (5′-GACTACHVGGGTATCTAATCC-3′) primers [34] amplified the V3–V4 region of the 16S rRNA gene. Fungal communities were amplified using the ITS7F (5′-GTGARTCATCGAATCTTTG-3′) and ITS4R (5′-TCCTCCGCTTATTGATATGC-3′) primers, targeting the ITS2 region [35]. For protists, the Euk1391F (5′-GTACACACCGCCCGTC-3′) and EukBr (5′- TGATCCTTCTGCAGGTTCACCTAC-3′) primers were used to amplify the V7-V9 region of the 18S rRNA gene [36]. Nematode communities were amplified using 1274F (5′- GACCCGTCTTGAAACACGGA-3′) and 706R (5′- GCCAGTTCTGCTTACC-3′), which target the D3-D5 region of the 28S rRNA gene [37, 38]. Polymerase chain reaction (PCR) conditions were optimized for each amplicon type. For bacterial 16S and nematode 28S rRNA gene amplifications, the thermocycling protocol consisted of an initial denaturation at 95 °C for 3 min, followed by 25 cycles of 95 °C for 30 s, 55 °C for 30 s, and 72 °C for 30 s, with a final extension at 72 °C for 5 min. For fungal ITS and protist 18S rRNA gene amplifications, the protocol included an initial denaturation at 94 °C for 5 min, followed by 25 cycles of 94 °C for 30 s, 57 °C for 30 s, and 72 °C for 30 s, concluding with a final extension at 72 °C for 5 min.

Indexing of amplicons was performed using the Nextera XT Index Kit v2 (Illumina, San Diego, CA, USA) under the following thermocycling conditions: 95 °C for 3 min; 8 cycles of 95 °C for 30 s, 55 °C for 30 s, and 72 °C for 30 s; and a final extension at 72 °C for 5 min. The indexed libraries were purified using AMPure XP beads (Beckman Coulter, Brea, CA, USA) and assessed for fragment size distribution using the Agilent TapeStation 4200 system. Equimolar concentrations of purified libraries were pooled and quantified using the Qubit dsDNA HS Assay Kit (Thermo Fisher Scientific). Sequencing was conducted on the Illumina MiSeq platform employing the MiSeq Reagent Kit v3 (600-cycle) (Illumina), generating paired-end reads of 2 × 300 bp.

Downstream sequence data processing was performed using Quantitative Insights into Microbial Ecology software (QIIME 2; https://qiime2.org/) [39]. Initially, generated raw paired end sequences were imported into QIIME 2 and demultiplexed using the qiime demux plugin followed by removing low-quality regions and chimeric sequences using the DADA2 plugin in QIIME2 [40]. Feature tables (amplicon sequence variants, ASVs) and feature data (representative sequences) were generated as resulting data from DADA2. Taxonomy was assigned to the ASVs with QIIME2 using a pre-trained Naive Bayes classifier [41] and compared against the SILVA v138 database for bacteria and nematodes, and UNITE v9.0 database for fungal, and PR2 v4.14.0 database for protist classification respectively [42–44]. Following ASV assignments, FUNGuild [45] was used to identify fungal trophic mode by grouping each guild into pathotrophs (plant and animal pathogens), saprotrophs (plant, soil and wood saprotroph), and symbiotrophs (arbuscular mycorrhizal fungi, ectomycorrhizal fungi, and endophytes). For taxa assigned to multiple modes (e.g., Pathotroph–Saprotroph–Symbiotroph), the first-listed or most ecologically plausible group was considered the dominant functional role [46]. To improve confidence, especially with highest trophic level (e.g., family) and multiple modes, we cross-referenced FUNGuild outputs with previous studies [25, 26] that reported consistent ecological roles under dryland conditions. Bacterial oligotrophs and copiotrophs were classified based on the RDP Classifier v2.12 integrated with the rrnDB database [47]. Taxa with mean rrn copy numbers ≥ 5 were classified as copiotrophs, and those with < 5 as oligotrophs, following established thresholds [48]. The functional groups of protists, such as consumers, phototrophs and pathothrophs, are classified on the basis of their feeding habits at the genus level [15, 49]. The protist taxa at the genus level are considered to have similar feeding modes and were grouped as either consumers or phototrophs. Similarly, all the trophic groups of nematodes, such as bacterivores, fungivores and omnivores, were collectively placed under consumers. While both the nematodes and protist consumers feed mainly on bacteria and other eukaryotes, phototrophic protists synthesize energy via photosynthesis, and other unassigned groups are defined as unknown.

### Statistical analyses

Unless otherwise noted, statistical analyses were performed in R software (R Core Team, 2020) and visualized using the “ggplot2” package (https://ggplot2.tidyverse.org) [50]. Before conducting soil organism community analyses, the ASV tables were rarefied to 4,485 sequences for bacteria, 10,526 for fungi, 1,079 for protists, and 412 for nematodes to ensure even sampling while retaining the maximum number of samples. Rarefaction curves were plotted for protists and nematodes to confirm that these rarefaction thresholds adequately capture species diversity (Fig. S1). Community composition across sites and rainfall treatments was visualised using principal coordinate analysis (PCoA) based on Bray–Curtis distance for each of the four taxa in the Phyloseq R package [51]. We analysed whether there was a statistical significance of site and treatment using permutational ANOVA (PERMANOVA) with the Adonis function in vegan package in R [51]. Additionally, distance-based redundancy analysis (dbRDA) using the Bray–Curtis distance matrix was performed to test the significance and importance of the environmental variables in explaining the variation in community composition across sites and treatments. Soil and environmental parameters were Total.C = total soil carbon, Total.N = total soil nitrogen, Total.P = total soil phosphorous, CN:ratio = soil C:N ratio, VR = vegetation richness, MAP = mean annual precipitation, OYR = one year rain, TMR = three month rain, and Temp = mean annual temperature (MAT). These analyses were performed using the capscale function of the vegan package [52].

### Co-occurrence network analysis

Multitrophic co-occurrence networks were constructed using a random matrix theory (RMT)-based approach implemented in the Molecular Ecological Network Analysis Pipeline (MENAP; http://ieg4.rccc.ou.edu/mena/) with default parameters [53, 54]. This method is widely used and has proven effective for constructing multitrophic co-occurrence networks [24, 55, 56], offering results that are comparable across ecological studies and consistent with our own previous analyses conducted at the same experimental site [24]. Prior to conducting the final network analysis, we compared network topology metrics between MENAP’s RMT-based Pearson correlation method and the compositionally aware SparCC approach with default parameters (via the iNAP platform; [57]) using the combined ASV dataset (n = 54 samples) that included bacteria and eukaryotes (fungi, protists, and nematodes). The 2 methods showed strong concordance across key network metrics (Table S2), consistent with previous evaluations by Hirano and Takemoto (2019) [48], thereby supporting the reliability and robustness of the RMT-based approach for constructing multitrophic ecological networks.

The RMT-based co-occurrence network method used in the MENAP addresses some of the key concerns in ecological network analysis, such as threshold selection, robustness to noise, high-dimensional data, and false positives, resulting in more reliable, replicable and meaningful ecological co-occurrence networks [58]. The raw ASV tables of bacteria and eukaryotes (fungi, protists, and nematodes) were merged into a table for co-occurrence network construction. Three multitrophic co-occurrence networks were constructed separately for each climatic condition: Semi-arid High CV (n = 18), Semi-arid Low CV (n = 18), and Arid (n = 18). To obtain robust associations between soil organisms, we used the MENAP-recommended optimal threshold of a Pearson correlation coefficient of 0.6 and false discovery rate (FDR)-corrected *p*-values at *p* < 0.001. This cutoff is widely applied and comparable across studies [24, 55, 56]. For each network, only ASVs detected in more than 50% of samples within that climatic group were retained, ensuring the inclusion of environmentally relevant and consistently occurring taxa. This group-specific filtering helps emphasize condition-specific associations while minimizing the influence of rare or sporadically detected ASVs.

The RMT-based approach can delineate separate modules, where each network was separated into modules by the fast-greedy modularity optimization. Each node in a module signifies an ASV and each edge signifies a significant (*p* < 0.05) pairwise association calculated based on the Pearson correlation coefficient. The network graph was represented using identified ASVs (nodes) with positive or negative interactions (edges). Positive interactions indicate that the abundances of the 2 associated ASVs changed following the same trend across different soil samples (i.e., they were positively correlated). Negative interactions indicate that the abundances of those ASVs changed following the opposite trend in different soil samples [59]. A modularity value measures the integrity of networks and is a fundamental characteristic of biological networks [54]. Each module in a network represents species with similar ecological niches that interact more frequently with each other than with species in other modules [53, 60]. Modularity is a key metric that gauges the extent to which a network is structured into well-defined modules, and it is a highly significant concept in ecology. Various factors can contribute to modularity, such as the specificity of interactions (such as predation or mutualism), diversity in habitat, resource partitioning, overlapping ecological niches, natural selection, convergent evolution, and phylogenetic relatedness. For comparison of network properties, empirical networks constructed using MENAP were evaluated against random networks generated by permuting the abundance data. The random networks retained the same number of nodes and edges but had randomized associations, allowing assessment of whether observed network metrics (e.g., modularity, clustering, average path length) differed from chance expectations.

To identify keystone taxa or functional groups, we analysed modular topological roles, which are based on the nodes’ roles within their respective modules. The topological role of each node (ASV) was defined by 2 parameters: within-module connectivity (Zi) and among-module connectivity (Pi) and the ZiPi scatter was plotted in Microsoft Excel. These ZiPi values were automatically calculated by MENAP based on the node’s degree distribution within and between modules, following the approach of Guimera & Amaral [61]. Within-module connectivity (Zi) and among-module connectivity (Pi) were calculated to identify the keystone ASVs. Previous research proposed threshold values of 2.5 for Zi and 0.62 for Pi, which were used to categorize the nodes into 4 groups [54, 61]. (i) Peripheral nodes (specialists) had low Zi (< 2.5) and low Pi (< 0.62) values. These nodes had only a few links and were almost always connected to the nodes within their own modules. (ii) Connectors (named generalists) had low Zi values (< 2.5) but high Pi values (> 0.62). These modules were highly connected with other modules. (iii) Module hubs (also named generalists) had high Zi values (> 2.5) but low Pi values (< 0.62). They were highly connected with many nodes in their own modules. (iv) Network hubs (named supergeneralists) had both high Zi (> 2.5) and Pi (> 0.62) values. Generalists (connectors, module hubs) and network hubs are the key organisms that play important roles in maintaining network stability. Additionally, the associations between module-based eigengenes and environmental variables were examined to elucidate the modules’ responses to environmental changes within the MENAP framework [53]. This analysis involved calculating Pearson correlation coefficients (r values) and their corresponding significance levels (*p* values).

To further explore the ecological relevance of dominant keystone taxa and functional groups, we performed Spearman correlation analyses in R. This non-parametric method was selected due to its robustness to non-normality and its ability to detect monotonic, potentially non-linear relationships, features particularly suitable for complex ecological datasets. Analyses targeted the relative abundances of dominant keystone taxa, such as fungal pathotrophs, saprotrophs, oligotrophic bacteria, and individual pathotrophic taxa, in relation to environmental drivers, thereby providing a taxon-specific perspective that extends beyond module-level patterns.

## Results

### Soil biotic communities differed among climatic conditions

**Fig. 2:**
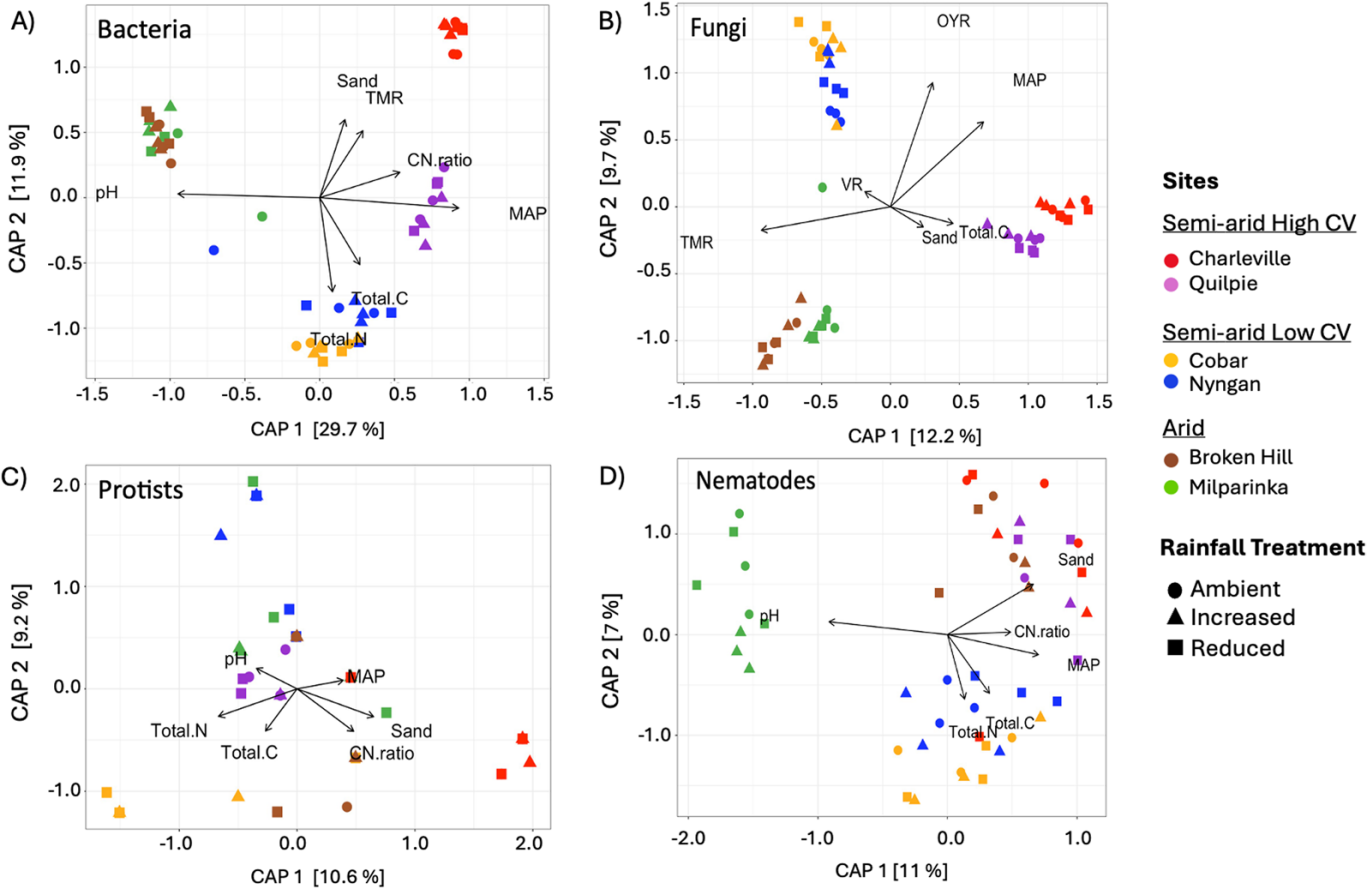
Distance-based redundancy analysis (db-RDA) biplots for bacteria **A**, fungi **B**, protists **C** and nematodes **D** including only environmental parameters that explained a significant amount of variability in community structure (arrows). The direction of the arrow indicates the direction of maximum change of that variable, whereas the length of the arrow is proportional to the magnitude of change to variables. MAP: mean annual precipitation; Total C: total soil carbon; Total N: total soil nitrogen; CN:ratio: carbon:nitrogen ratio; VR: vegetation richness; OYR: one-year rainfall; TMR: three-month rainfall; Temp.: Temperature

Bacterial and eukaryotic community analyses across sites and rainfall treatments indicated that the variation between datasets was predominantly explained by climatic conditions (PERMANOVA, *p* < 0.001; Fig. S2), with no effect of rainfall treatment for any of the four groups, either overall or within individual climatic conditions (Table S3). Similarly, community diversity measured using Shannon and Chao1 indices was significantly influenced by climatic condition but not by rainfall treatment (Table S3). Both bacterial and fungal communities displayed distinct clustering patterns, with Arid (Broken Hill and Milparinka), Semi-arid High CV (Cobar and Nyngan), and Semi-arid High CV (Charleville and Quilpie) forming separate clusters (Fig. S2). However, such clear clustering patterns were not observed for the protist and nematode communities. Distance-based redundancy analysis (dbRDA) was used to examine the relationships between biotic communities and environmental variables across sites and rainfall treatments. PERMANOVA results indicated statistically significant associations (*p* < 0.05) between environmental variables and the biotic communities. All groups showed strong correlations with mean annual precipitation (MAP) (bacteria, fungi and nematodes: *p* < 0.001; protists: *p* < 0.005). Except for fungi, soil pH was strongly associated with all other groups (bacteria and nematodes: *p* < 0.001; protists: *p* < 0.01), as were total soil carbon and nitrogen contents (*p* < 0.005) (Fig. 2). Notably, MAP was more closely associated with semi-arid conditions, whereas soil pH showed stronger associations with arid conditions.

### Arid multitrophic co-occurrence network exhibited stronger clustering compared to semi-arid networks

Our findings revealed that the Arid network was the most complex, exhibiting the greatest number of nodes and edges (148 nodes, 546 edges), followed by the Semi-arid Low CV (130 nodes, 302 edges) and Semi-arid High CV (115 nodes, 315 edges) networks (Table 1). Interestingly, only the Arid network showed more positive associations than negative associations. Thus, the ratio of positive to negative links in the Arid network (1.4) was greater than that in the Semi-arid Low CV (0.86) and Semi-arid High CV (0.56) networks. Compared with those of the Semi-arid High CV (8.081, 0.139) and Arid (7.622, 0.161) networks, the average degree (avgk) and average clustering coefficient (avgCC) of Semi-arid Low CV had the lowest values (4.646, 0.08). However, the Arid network had the lowest average path distance (GD; 3.177) relative to the 2 Semi-arid networks (3.553 and 3.429). This suggests that the nodes of the Arid network were more closely clustered than those of the two other networks. In addition, the modularity value of empirical networks was much higher than that of random networks, as observed in the case of Arid network. Similarly, larger differences can also be observed with the average path distance (GD) which is higher in the empirical network than in the random network (Table 1).

**Table 1:** Empirical and random network properties of Semi-arid Low CV, High CV, and Arid multitrophic co-occurrence networks

Empirical network properties	Semi-arid low CV	Semi-arid high CV	Arid
Number of nodes	130	124	148
Number of edges Positive Negative Ratio ±	302 140 162 0.86	501 181 320 0.56	546 319 227 1.4
Average degree (avgK)	4.646	8.081	7.622
Average clustering coefficient (avgCC)	0.080	0.139	0.161
Average path distance (GD)	3.553	3.429	3.177
Modularity (no. of modules)	0.465 (12)	0.296 (8)	0.434 (8)
*Random networks properties*			
Average clustering coefficient (avgCC) (± SD)	0.066 (± 0.011)	0.211 (± 0.019)	0.123 (± 0.013)
Average path distance (GD) (± SD)	3.181 (± 0.056)	2.624 (± 0.035)	2.788 (± 0.042)
Modularity (± SD)	0.400 (± 0.008)	0.246 (± 0.006)	0.297 (± 0.007)

**Fig. 3:**
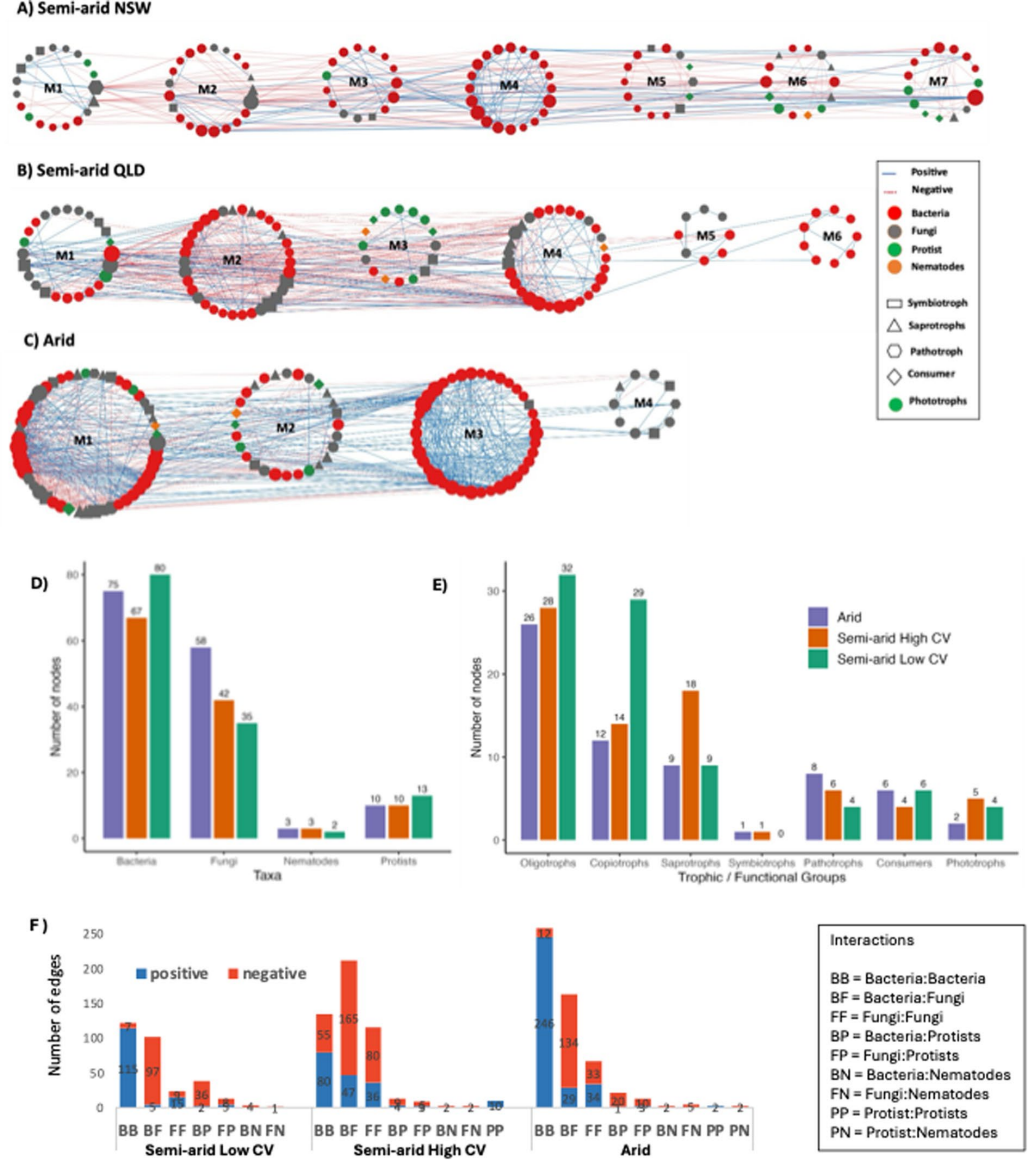
Multitrophic co-occurrence networks across the three climatic conditions. **A** Semi-arid Low CV, **B** Semi-arid High CV, and **C** Arid. The networks were separated into modules by the fast-greedy modularity optimization, only modules with five or more nodes were included. Each node represents different prokaryotic or eukaryotic families, and the size of the node is proportional to the number of connections (degree). A blue edge indicates a positive interaction between two individual nodes, while a red dotted edge indicates a negative interaction. The shape of the nodes represents their trophic functional mode. Bar plots summarize **D** the number of nodes per climatic condition, **E** their trophic or functional groups, including bacteria (oligotrophs, copiotrophs), fungi (saprotrophs, symbiotrophs, pathotrophs), and protists (consumers, phototrophs) and **F** the number of positive (blue) and negative (red) inter- and intra-domain links for each condition

In the Semi-arid Low CV and High CV networks, seven and six modules (with ≥ 5 nodes) were respectively obtained, whereas the Arid network had only four such modules (Fig. 3). The modules within the Arid network comprised 3 large modules that were closely connected, whereas the Semi-arid High CV network had four larger modules. In contrast, the Semi-arid Low CV network had nodes that were more evenly distributed across modules. Most of the modules included all the trophic groups, with only a few represented by a single trophic group (such as the bacteria-dominated Semi-arid Low CV M3 and Arid M3 modules).

### Multitrophic associations differ across semi-arid and arid climatic conditions

Comparison of inter-group networks revealed that bacteria had the highest number of nodes, followed by fungi, protists, and nematodes, with node counts varying among the networks (Fig. 3D). The Semi-arid Low CV network exhibited the highest bacterial (80 nodes) and protist (13 nodes) counts, but relatively fewer fungal nodes (35 nodes). In contrast, Arid and Semi-arid High CV networks had higher fungal node counts (58 and 42 nodes, respectively) and slightly higher nematode counts (3 nodes each). Examination of trophic group nodes revealed that pathotrophs were most abundant in the Arid network (8 nodes), while saprotrophs peaked in the Semi-arid High CV network (18 nodes). The lowest pathotroph node count (4 nodes) occurred in the Semi-arid Low CV network, and lowest phototroph node count (2 nodes) was observed in the Arid network (Fig. 3; E). Notably, bacterial oligotrophs (32 nodes) and copiotrophs (29 nodes) were highest in the Semi-arid Low CV network, whereas the Arid and Semi-arid High CV networks exhibited higher oligotrophs-to-copiotrophs ratio (26:12 nodes and 28:14 nodes, respectively), suggesting that oligotrophs dominated bacterial communities under these conditions (Fig. 3; E).

Additionally, the study compared the number of positive and negative edges (associations) within and between biotic groups (Fig. 3; F). The Semi-arid Low CV and Arid networks were dominated by bacteria-bacteria associations (BB; 40% and 47.25%, respectively) whereas the Semi-arid High CV network was mainly dominated by bacteria-fungi associations (BF; 42%). Fewer associations were observed with protists and nematodes. Protists showed more associations with bacteria and fungi in Semi-arid Low CV (BP; 12.5% and FP; 4.3%) than Arid (BP; 3.8% and FP; 2.3%) and Semi-arid High CV (BP; 2.5% and FP; 1.7%). Nematodes showed only a few associations with bacteria (BN) and fungi (FN), together representing less than 1.5% of the total number of associations across all three networks (Fig. 3; F). Additionally, the bacteria-bacteria associations presented the highest positive-to-negative link ratio, while bacteria-fungi had the lowest positive-to-negative link ratio in all three networks.

### The keystone taxa are predominantly fungal pathothrophs, saprotroph and oligotrophic bacteria

**Fig. 4:**
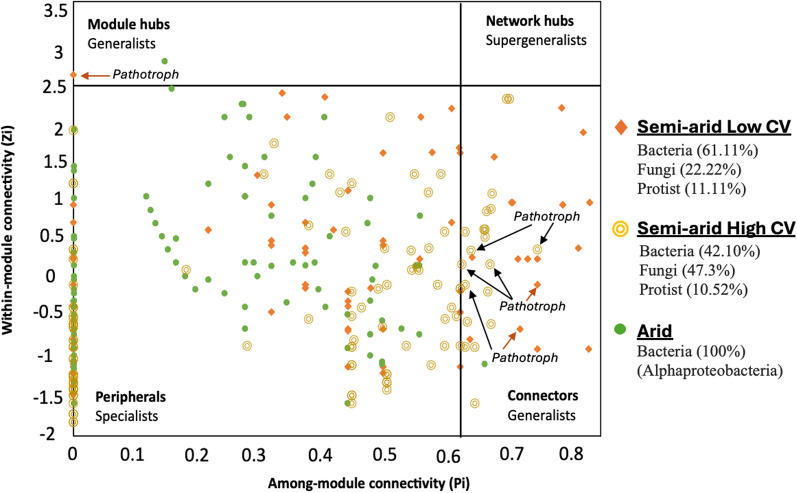
Combined ZiPi scatter plot of network node topology for Semi-arid Low CV, Semi-arid High CV and Arid networks. Arrows indicate the identified fungal pathotroph groups. Detailed taxonomic information for nodes in module hubs and connectors can be found in Table S4. The taxa (nodes) in the table match the order shown in the figure from the top (module hubs) to bottom (connectors), for each network separately

The ZiPi-plot was used to illustrate the topological roles of nodes, effectively identifying key populations or functional groups within each network. The nodes were classified into four categories on the basis of their values of Zi and Pi, which were peripherals, connectors, module hubs, and network hubs (Fig. 4). In Semi-arid Low CV, there were a total of 17 connectors and one module hub (identified as genus *Alternaria*, Ascomycota), while semi-arid High had 19 connectors but no module hub. The connectors associated with Semi-arid Low CV were mostly dominated by Actinobacteria (29.41%) followed by Ascomycota (17.64%), Proteobacteria (17.64%), and two protist consumers (Rhizaria, 11.76%) (Table S4). In contrast, the connectors in Semi-arid High CV were dominated by Ascomycota (47.36%), followed by Actinobacteria (21.05%) and Chloroflexi (10.52%), alongside two protist phototrophs (Archaeplastida and Stramenophiles, 10.52%). On the other hand, Arid network had only one connector and one module hub, both of which were Alphaproteobacteria of the genera *Balneimonas* and *Pseudonocardia*. Interestingly, across all networks, the identified keystone taxa are mostly oligotrophic bacteria, along with fungal pathotrophs followed by fungal saprotrophs, which collectively function as major key connectors of belowground multitrophic groups (Table S4).

### Correlation between network modules, keystone groups, and environmental variables

Correlations between network modules and environmental variables revealed significant relationships with vegetation and soil attributes. In Semi-arid Low CV, Module M3 was positively associated with phosphorus content (r = 0.6, P = 0.008), while Module M7 and M2 showed negative (r = -0.05, P = 0.02) and positive (r = 0.49, P = 0.04) correlations with standing biomass (SB), respectively (Table S5). In Semi-arid High CV, Module M2 was positively correlated with vegetation richness (VR) (r = 0.6, P = 0.009), whereas Module M6 was negatively associated with SB (r = -0.6, P = 0.009). For the Arid network, Module M3 was negatively correlated with pH (r = -0.6, P = 0.008) and SB (r = -0.6, P = 0.003), and Module M2 with VR (r = -0.5, P = 0.02) (Table S5). In general, bacteria-dominated modules were negatively correlated with SB, whereas fungal symbiotroph and saprotroph-dominated modules were positively associated with SB and VR (Table S5). Additionally, protist-dominated modules correlated positively with phosphorus. These findings underscore the distinct environmental responses of different submodules across dryland networks, with key abiotic factors impacting specific groups.

**Fig. 5:**
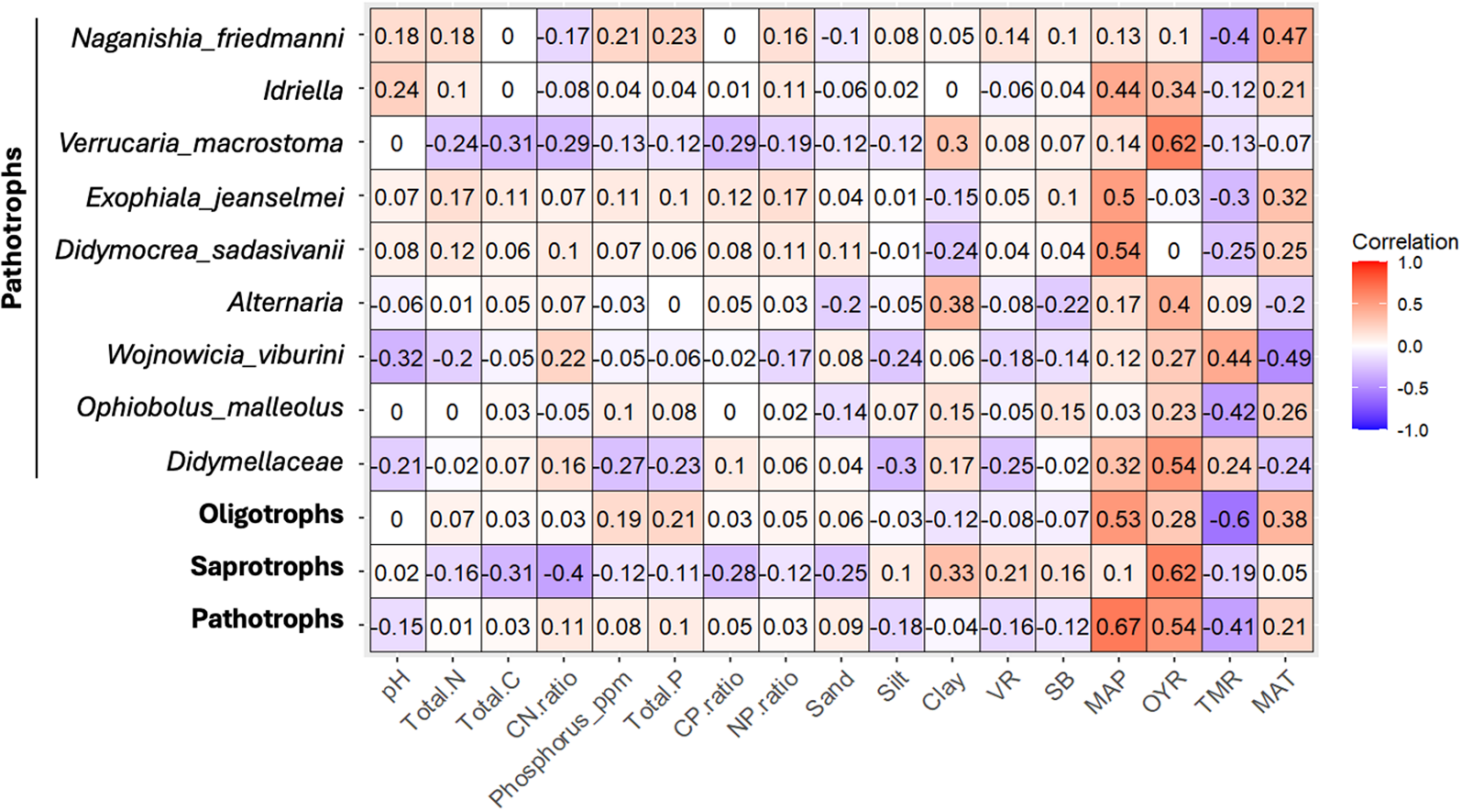
Spearman correlations between environmental factors and the relative abundances of combined keystone fungal pathotrophs, saprotrophs, oligotrophic bacteria, and individual pathotroph taxa (italicized genus or species). Correlation values are shown as either positive or negative, with color intensity indicating the strength of the relationship: darker brown for stronger positive correlations and darker blue for stronger negative correlations. Only significant correlations are highlighted. Environmental variables include pH, Total C (carbon), N (nitrogen), P (phosphorus), Phosphorous parts per million (ppm), C:N, C:P, N:P, VR (vegetation richness), SB (standing biomass), MAP (mean annual precipitation), OYR (one-year rainfall), TMR (three-month rainfall), and MAT (mean annual temperature)

Additionally, the influence of environmental variables on the dominant identified keystone trophic groups and individual fungal pathotrophic taxa were assessed. Keystone taxa such as bacterial copiotrophs and protists were excluded due to their minimal presence, with the analysis focusing solely on the dominant groups. The results revealed that most keystone taxa, including fungal pathotrophs and bacterial oligotrophs, were strongly positively associated with MAP, OYR and MAT. Fungal saprotrophs, on the other hand, were primarily correlated with OYR. In contrast, TMR exhibited strong negative associations with the identified keystone groups (Fig. 5).

## Discussion

Altered rainfall patterns resulting from climate change can have detrimental consequences for biodiversity in semi-arid and arid ecosystems, impacting organisms across all trophic levels [13, 55, 62]. Community composition of four key soil groups, i.e., bacteria, fungi, protists, and nematodes, were significantly different among three distinct climatic conditions, specifically Arid, Semi-arid High CV and Semi-arid Low CV. Notably, protists and nematodes, often underrepresented in dryland studies, can be affected by varying rainfall and aridity to the same extent as bacterial and fungal communities. Consistent with our previous findings [24], rainfall treatment effects were not observed on any of the studied group. Instead, community was largely shaped by regional environmental factors. In particular, mean annual precipitation (MAP) and soil properties such as pH, soil total carbon and nitrogen were key drivers of belowground community structure. The observed stability across rainfall treatments likely reflects the dominance of stress-adapted taxa and overall higher resilience of these dryland communities to drought manipulations. These results are in line with previous studies, illustrating how multiple abiotic factors drive belowground communities, with differences in aridity associated with annual rainfall being a major factor [8, 14, 55].

Aridity is a well-documented driver of soil microbiome network structure [8], and our findings extend this understanding to multitrophic levels. The Arid multitrophic network was the most complex, exhibiting the greatest number of nodes and edges, followed by the Semi-arid Low CV and Semi-arid High CV networks. Specifically, the analysis showed that increasing aridity led to a higher proportion of significant positive associations among bacterial nodes, suggesting enhanced microbial cohesion under environmental stress. Previous research has similarly shown an increase in bacterial abundance and positive associations with increasing aridity [4, 8]. The higher modularity observed in Arid and Semi-arid Low CV networks suggests more strongly compartmentalized multitrophic networks, a pattern typically associated with greater ecological stability. This is further supported by varying responses of different modules to environmental factors, suggesting a significant impact on the constituents of certain members of some submodules, particularly those associated with soil phosphorous and vegetation. Such compartmentalization likely enhance network resilience by structuring taxa into functional units that respond independently to environmental pressure [63]. Conversely, the Semi-arid High CV network displayed a higher ratio of bacteria–fungi (BF) associations, potentially reflecting more dynamic cross-kingdom interactions in regions with greater rainfall variability and indicating a less compartmentalized, more adaptable microbial community [21, 64]. A key aspect of this study is the inclusion of protists and nematodes, which have often been overlooked in network studies despite their critical trophic roles [7]. Protists were notably more integrated into the Semi-arid Low CV network, with a higher frequency of bacteria–protist and fungi–protist associations. These patterns suggest enhanced microbial grazing and energy transfer in more climatically stable semi-arid environments [65]. In contrast, protist connectivity was much lower in the Arid and Semi-arid High CV networks, likely reflecting stress-induced reductions in prey availability or environmental conditions unfavorable for protist proliferation, suggesting diminished top-down regulation [10]. Although nematodes were less abundant and less connected across networks, they are known to promote certain stress-adapted bacterial phyla and strengthen their connectance, including Actinobacteria, Chloroflexi, and Proteobacteria [11], which were prevalent in our data. Their limited connectivity may reflect more specialized feeding preferences or underrepresentation in co-occurrence networks.

Topological roles analysis further revealed that Semi-arid networks have a higher abundance of putative keystone taxa (module hubs and connectors) compared to the Arid network, indicating greater multitrophic interconnectedness in less arid ecosystems. Additionally, the reduced number of keystone taxa observed in the Arid network may correspond to fewer but larger modules, a pattern also noted in our study and consistent with previous finding [66]. Dominant keystone taxa included oligotrophic bacteria and fungal pathotrophs and saprotrophs, belonging to phyla Actinobacteria, Alphaproteobacteria, Chloroflexi and Ascomycota. This supports the notion that arid conditions promote slow-growing, drought-adapted microbial taxa, leading to the co-occurrence of oligotrophic assemblages [8]. The higher dominance of keystone fungal pathotrophs highlights a significant ecological concern, as many such taxa, including *Alternaria sp., Ophiobolus malleolus,* and Brotryosphaeriaceae family, are opportunistic pathogens that proliferate under plant stress [26, 45] [67]. This aligns with broader observations of rising soil-borne fungal pathogens under climate change, particularly driven by rising temperatures [25, 26, 68]. While our study also identified temperature correlations, variation in mean annual rainfall emerged as the primary driver influencing these keystone groups, consistent with overall community composition trends. Moreover, the rising dominance of fungal pathotrophs coincided with the absence of keystone protist consumers in more arid conditions, suggesting potential shifts in trophic interactions under increasing aridity. Additionally, higher keystone saprotrophs abundance underscores their key role in nutrient redistribution; these fungi function as nutrient ‘miners,’ decomposing complex organic substances to acquire energy and nutrients, which may enhance their persistence under stress [69]. Some pathotrophs may function primarily as saprotrophs, while certain saprotrophic fungi, such as *Idriella* and Eurotiomycetes, can also switch to act as opportunistic pathogens under stress [69]. Given ongoing climate change, the prevalence of opportunistic fungal pathogen is expected to rise as environmental stressors intensify [68].

In addition, keystone protist functional groups, such as consumers and phototrophs (Rhizaria, Archaeplastida, and Stramenopiles), showed close association with bacterial and fungal communities. Consistent with previous observations, protist consumers were more dominant in the bacteria-rich Semi-arid Low CV network [10], whereas phototrophs were more prominent in the fungal-dominated Semi-arid High CV. Protist consumers, such as Filosa-Thecofilosea and Filosa-Sarcomonadea within Rhizaria, likely exert strong top-down control on bacterial populations, promoting microbial turnover and nutrient mineralization, particularly under stable semi-arid conditions [13]. In contrast, the presence of keystone phototrophic protists in more arid conditions, together with higher saprotroph abundance, may indicate indirect support for fungal saprotrophic activity under stress [10, 13]. Nematodes were less prominent in multitrophic networks, reinforcing the concept that while smaller organisms like bacteria and protists actively drive many soil processes, larger organisms such as nematodes primarily regulate broader ecosystem functions rather than directly engaging in numerous microbial interactions [70]. Overall, our findings emphasize the importance of dominant bacterial and fungal groups, particularly oligotrophs and pathotrophs, which play crucial roles in shaping soil food web complexity and responses to climate change.

This study offers valuable insights into soil food web complexity and trophic interactions; however, it is important to acknowledge some potential limitations. Correlation-based co-occurrence networks provide a simplified view and may not fully represent real-world soil food webs [71]. Such networks can produce spurious associations and may not capture the complete architecture and connectedness of soil ecosystems. Nonetheless, they remain useful for estimating species relationships and understanding the impact of network complexity on biodiversity and ecosystem functioning [8, 22, 53, 55, 72]. Furthermore, sequencing approaches such as metabarcoding may underrepresent larger soil invertebrates like nematodes, partly due to primer biases and variable DNA extraction efficiencies. While some studies have successfully applied metabarcoding to estimate soil invertebrate diversity [12, 55, 73], challenges remain in fully capturing the diversity and trophic roles of nematodes and protists using current protocols. Databases like NemaBase offer promising tools to improve nematode identification and ecological interpretation, but further advances are needed. Future research should focus on integrating improved reference databases, multi-marker metabarcoding, complementary multi-omics techniques, and experimental validation will be essential to deepen understanding of soil multitrophic dynamics in dryland ecosystems.

## Conclusion

Our findings reveal that increasing aridity and interannual rainfall variability restructure soil multitrophic networks in dryland ecosystems, promoting positive associations among oligotrophic bacteria and fostering communities dominated by stress-adapted taxa. Fungal pathotrophs and bacterial oligotrophs emerge as key drivers of belowground food webs, underscoring their critical roles in ecosystem functioning under climate change. The concurrent rise of fungal pathotrophs, likely linked to declines in protist consumers, signals a growing threat to ecosystem resilience. Integrating multitrophic perspectives will be essential in future research to better predict and manage emerging threats, such as the proliferation of soil-borne pathogens, and to develop management strategies aimed at safeguarding dryland ecosystem functioning.

## Supplementary Information

Supplementary file 1.

## Data Availability

The raw sequencing data generated in this study are available in the NCBI Sequence Read Archive (SRA) under BioProject accession number PRJNA1241245 (https://www.ncbi.nlm.nih.gov/bioproject/PRJNA1241245). Associated metadata, including environmental variables, soil physicochemical properties, and plant richness and standing biomass, and ASV tables used in network construction, along with network properties and cytoscape file generated using both RMT and SparCC methods, are publicly accessible via Figshare at 10.6084/m9.figshare.28672310.
