# Supplementary material for: Oligotrophic bacteria and pathotrophic fungi moderate multitrophic interactions in semi-arid and arid environments
Source: Environ Microbiome. 2025 Nov 19;20:146. doi: 10.1186/s40793-025-00788-1 (PMC12628942; doi:10.1186/s40793-025-00788-1)
Supplement: Supplementary file 1 [file 40793_2025_788_MOESM1_ESM.docx]

**Title: Oligotrophic bacteria and pathotrophic fungi moderate multitrophic interactions in semi-arid and arid environments.**

***Supplementary Materials***

*Table S1.* *List of dryland field sites including site name, ecosystem type, long-term mean annual precipitation (MAP, in mm year−1), inter-annual rainfall variability (Coefficient of Variation, CV), mean annual temperature (MAT, in ºC), aridity indices (AI), one year rainfall (OYR), three-month rainfall (TMR) measured in the control plot. Long term mean annual rainfall and temperature based on WorldClim2 (1970–2000).*

| **Site** | **Ecosystem Type** | **Latitude/ Longitude** | **Dominant Species/Vegetation** | **Soil Description** | **CV** | **pH** | **MAP** | **OYR** | **TMR** | **MAT** | **AI** |
| --- | --- | --- | --- | --- | --- | --- | --- | --- | --- | --- | --- |
| **Broken Hill** | Arid | 32.0°S, 141.6°E | Maireana pyramidata | Brown calcareous sand | 0.28 | 5.42 | 238 | 181 | 91 | 18.3 | 0.11 |
| **Milparinka** | Arid | 29.6°S, 141.7°E | Astreblea lappeacea, A. pectinata, and Abutilon halophilum | Grey ‘cracking’ clay | 0.52 | 6.07 | 227 | 189 | 67.6 | 20.3 | 0.12 |
| **Charleville** | Semi-arid High CV | 26.4°S, 146.2°E | Eremophila gilesii (native invasive woody scrub) | Red sandy, porous | 0.53 | 6 | 349 | 439.6 | 58.8 | 18.8 | 0.25 |
| **Quilpie** | Semi-arid High CV | 26.6°S, 144.6°E | Eragrostis setifolia, Sclerolaena species, and Abutilon halophilum | Hard red clay, gravelly | 0.57 | 6.2 | 390 | 343.4 | 27.6 | 21.9 | 0.16 |
| **Cobar** | Semi-arid  Low CV | 31.8°S, 145.6°E | Erodium crinitum, Helipterum species, Medicago species, Ptilotus species, and Austrostipa scabra | Lowland floodplain red soil, clay with gravel | 0.27 | 5.93 | 462 | 401.4 | 32.4 | 20.9 | 0.25 |
| **Nyngan** | Semi-arid  Low CV | 31.7°S, 146.6°E | Calotis lappulacea, Erodium crinitum, Austrostipa scabra, Helipterum species, and Medicago species | Upland floodplain red soil, clay with gravel | 0.31 | 5.99 | 441 | 493.6 | 72.2 | 18.6 | 0.3 |


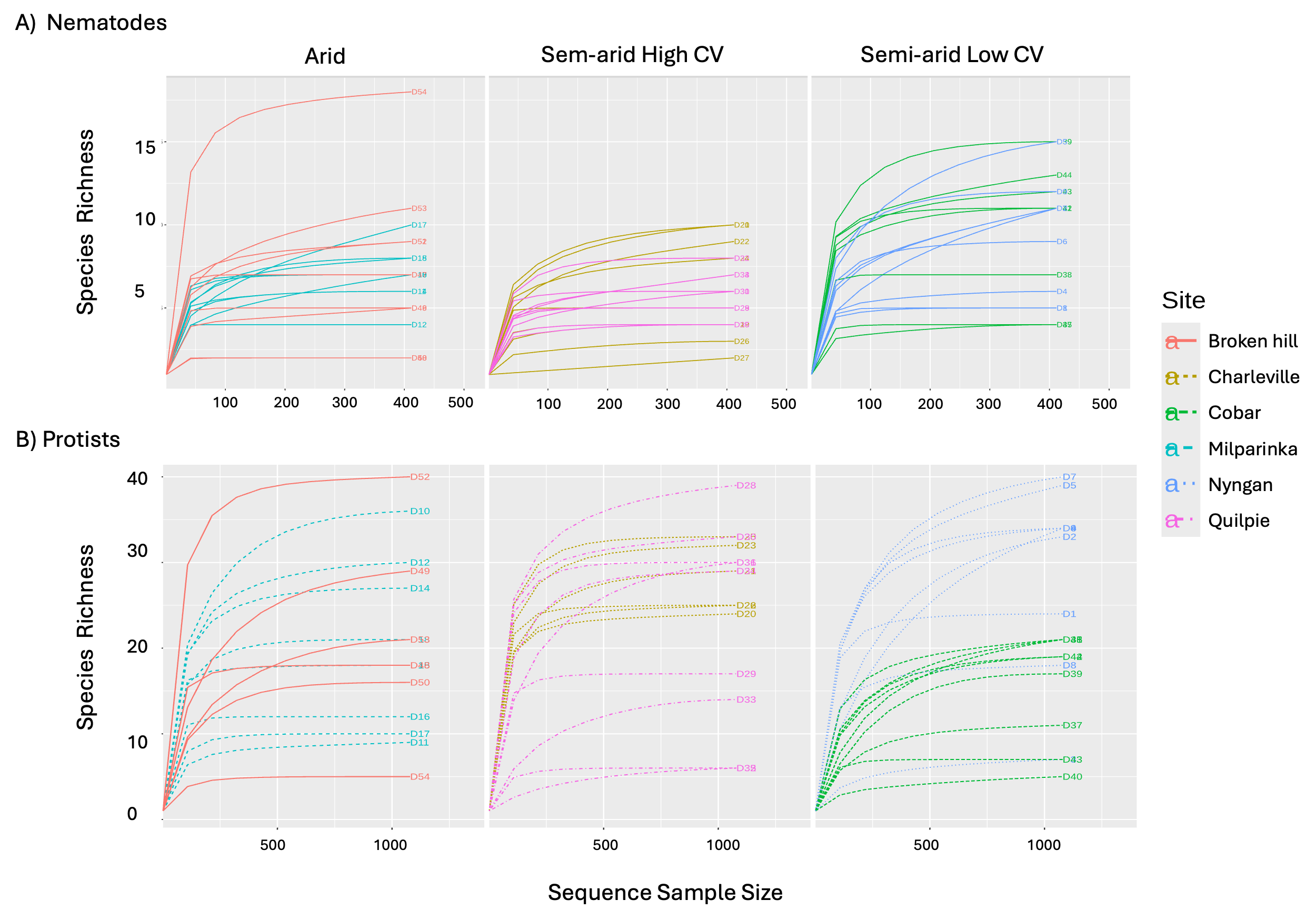


*Fig. S1* *Rarefaction curves of nematode (A) and protist (B) communities across climatic conditions: Arid (Broken Hill and Milparinka), Semi-arid High CV (Charleville and Quilpie), and Semi-arid Low CV (Nyngan and Cobar). The curves demonstrate that the observed rarefied values provide a balanced and representative estimate of species diversity within each group.*


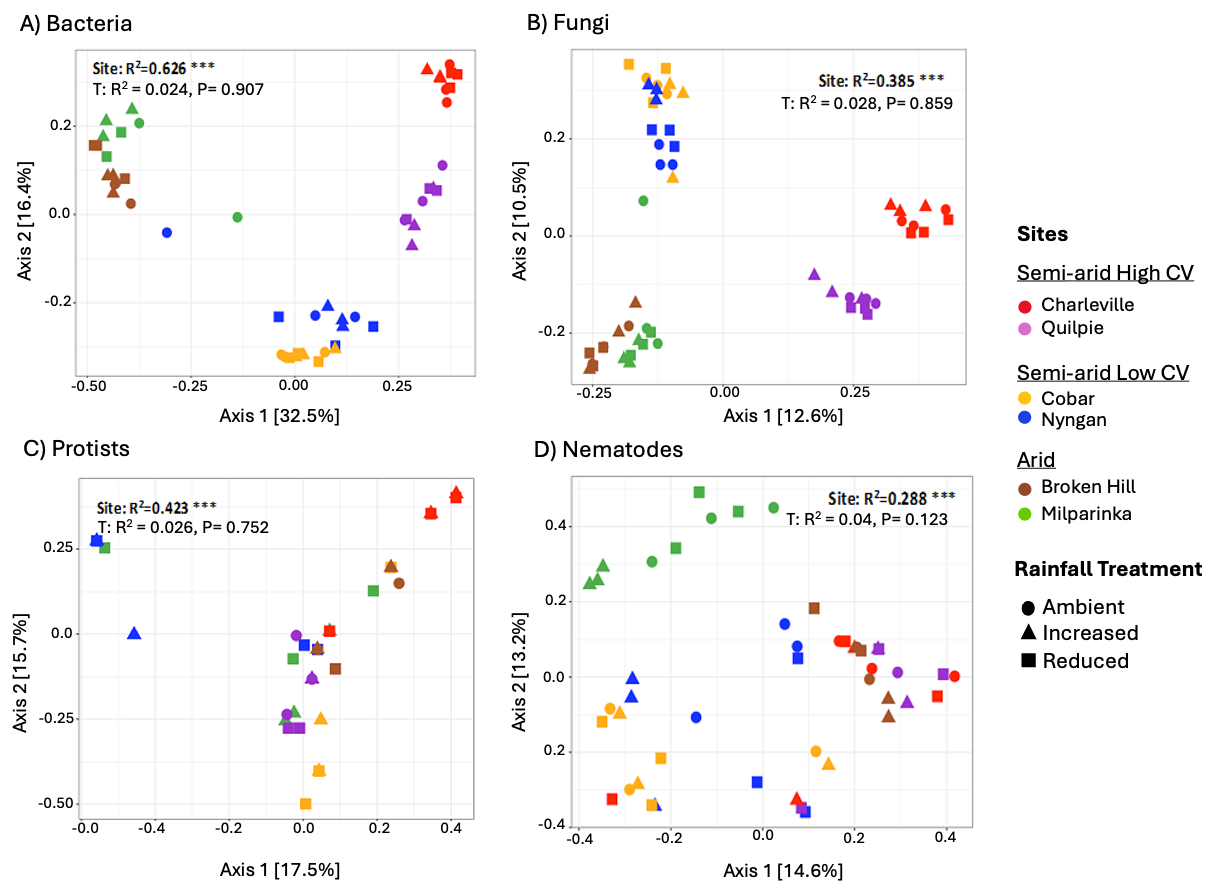


*Fig. S2 Plots showing Principal Coordinates Analyses (PCoA) based on Bray–Curtis distance matrices for (A) Bacterial, (B) Fungal, (C) Protist, and (D) Nematode community structure across Sites and Rainfall treatments. Statistics for significant PERMANOVA results testing the effect of Site, Rainfall and their interactions on composition inserted. *Significance codes: *** = <0.001; ** = <0.01; * = 0.05.*

*Table S2.* *Comparison of multitrophic cooccurrence network properties derived from RMT-Pearson correlation and SparCC of combined ASV dataset (n = 54 samples) that included bacteria and eukaryotes (fungi, protists, and nematodes).*

| **Network metrics** | **RMT-Pearson Correlation** | **SparCC** |
| --- | --- | --- |
| Number of Nodes | 98 | 75 |
| Number of edges | 288 | 197 |
| Positive (%) | 60 | 65.5 |
| Negative (%) | 40 | 34.5 |
| Average number of neighbors | 5.878 | 5.253 |
| Network diameter | 7 | 7 |
| Network radius | 1 | 1 |
| Network density | 0.03 | 0.035 |
| Clustering coefficient | 0.133 | 0.201 |
|  |  |  |
| **Trophic group distribution (%)** |  |  |
| Bacteria | 67.06 | 67.09 |
| Fungi | 28.24 | 25.32 |
| Protist/Nematodes | 4.71 | 7.59 |

*Table S3. PERMANOVA results based on Bray–Curtis dissimilarity showing the effects of rainfall treatment on bacterial, fungal, protist, and nematode community composition within each climatic type. Reported values include R², F, and P values. Additionally, results from ANOVA tests on Chao1 richness and Shannon diversity indices are provided, showing the effects of climate type and rainfall treatment within each climate category. For ANOVA, F and P values are reported. Statistically significant results (P < 0.05) are indicated in bold.*

| Climate type | **Rainfall treatment** | | |  |
| --- | --- | --- | --- | --- |
|  | PERMANOVA | | |  |
| **Bacteria** | **R^2^** | **F** | **P value** |  |
| Semi-arid Low | 0.1492 | 0.9645 | 0.5012 |  |
| Semi-arid High | 0.08954 | 0.7375 | 0.655 |  |
| Arid | 0.19466 | 0.9669 | 0.4642 |  |
|  |  |  |  |  |
| **Fungi** | R^2^ | F | P value |  |
| Semi-arid Low | 0.09427 | 0.7806 | 0.7467 |  |
| Semi-arid High | 0.10502 | 0.8801 | 0.5574 |  |
| Arid | 0.10532 | 0.8829 | 0.6418 |  |
|  |  |  |  |  |
| **Protists** | R^2^ | F | P value |  |
| Semi-arid Low | 0.11799 | 1.0033 | 0.3912 |  |
| Semi-arid High | 0.12624 | 0.8669 | 0.5019 |  |
| Arid | 0.06886 | 0.4807 | 0.8889 |  |
|  |  |  |  |  |
| **Nematodes** | R^2^ | F | P value |  |
| Semi-arid Low | 0.08921 | 0.6856 | 0.6765 |  |
| Semi-arid High | 0.14352 | 1.2568 | 0.2735 |  |
| Arid | 0.12044 | 1.027 | 0.436 |  |
|  |  |  |  |  |
| **Climate** | | | | |
|  | **Shannon** | | **Chao1** | |
|  | F | P value | F | P value |
| **Bacteria** | **27.34** | **3.28e-08 ***** | **12.47** | **6.18e-05 ***** |
| **Fungi** | 1.89 | 0.162 | **3.38** | **0.0418 *** |
| **Protists** | **6.016** | **0.00478 **** | 0.23 | 0.795 |
| **Nematodes** | **3.943** | **0.0257 *** | **5.944** | **0.00483 **** |
|  |  |  |  |  |
|  |  |  |  |  |
| **Rainfall treatment** | | | | |
|  | **Shannon** | | **Chao1** | |
| **Bacteria** | F | P value | F | P value |
| Semi-arid Low | 0.774 | 0.485 | 3.034 | 0.0893 |
| Semi-arid High | 0.314 | 0.7165 | 1.445 | 0.267 |
| Arid | 0.012 | 0.988 | 0.216 | 0.81 |
|  |  |  |  |  |
| **Fungi** | F | P value | F | P value |
| Semi-arid Low | 2.74 | 0.06477 | 1.385 | 0.281 |
| Semi-arid High | 0.618 | 0.552 | 0.50292 | 0.7777 |
| Arid | 0.061 | 0.9268 | 0.153 | 0.859 |
|  |  |  |  |  |
| **Protists** | F | P value | F | P value |
| Semi-arid Low | 0.848 | 0.448 | 1.405 | 0.276 |
| Semi-arid High | 2.18 | 0.3362 | 0.315 | 0.735 |
| Arid | 0.04 | 0.961 | 0.185 | 0.833 |
|  |  |  |  |  |
| **Nematodes** | F | P value | F | P value |
| Semi-arid Low | 0.378 | 0.692 | 0.095 | 0.91 |
| Semi-arid High | 1.2807 | 0.2778 | 3.003 | 0.08 |
| Arid | 0.319 | 0.731 | 1.041 | 0.377 |

*Table S4. Keystone groups identified using the ZiPi plot, showing their classification at the highest taxonomic level and their associated trophic modes.*

| **Group** | **Phylum** | **taxa** | **Trophic group** |
| --- | --- | --- | --- |
|  |  |  |  |
|  |  | **Semi-arid Low CV** |  |
| **Module Hub (Generalist)** |  |  |  |
| Fungi | Ascomycota | *Alternaria (genus)* | **Pathothroph** |
|  |  |  |  |
| **Connectors (Generalist)** |  |  |  |
| Bacteria | Actinobacteria | Actinomycetales (order) | Oligotroph |
| Bacteria | Actinobacteria | Frankiaceae (family) | Oligotroph |
| Bacteria | Actinobacteria | *Geodermatophilus (genus)* | Oligotroph |
| Bacteria | Actinobacteria | Microbacteriaceae (family) | Oligotroph |
| Bacteria | Actinobacteria | *Amycolatopsis (genus)* | Oligotroph |
| Bacteria | Actinobacteria | Solirubrobacteraceae (family) | Oligotroph |
| Bacteria | Chloroflexi | Thermomicrobia (class) | Oligotroph |
| Bacteria | Gemmatimonadetes | Gemmatimonadetes (class) | Oligotroph |
| Bacteria | Deltaproteobacteria | Cystobacteraceae (family) | Copiotroph |
| Bacteria | Alphaproteobacteria | Rhizobiales (order) | Oligotroph |
| Bacteria | Alphaproteobacteria | Acetobacteraceae (family) | Oligotroph |
| Fungi | Ascomycota | *Idriella (genus)* | Saprotroph |
| Fungi | Ascomycota | Didymellaceae (family) | **Pathothroph** |
| Fungi | Ascomycota | *Wojnowicia_viburni (species)* | **Pathothroph** |
| Fungi | Basidiomycota | *Naganishia (genus)* | Saprotroph |
| Protist | Cercozoa | Filosa-Thecofilosea | consumer |
| Protist | Cercozoa | Filosa-Sarcomonadea | consumer |
|  |  |  |  |
|  |  | **Semi-arid High CV** |  |
| **Connectors (Generalist)** |  |  |  |
| Bacteria | Actinobacteria | Georgenia (genus) | Oligotroph |
| Bacteria | Actinobacteria | Nocardiacea (family) | Oligotroph |
| Bacteria | Actinobacteria | *Actinomycetospora (genus)* | Oligotroph |
| Bacteria | Actinobacteria | Gaiellaceae (family) | Oligotroph |
| Bacteria | Chloroflexic | Ktedonobacteria (class) | Oligotroph |
| Bacteria | Chloroflexic | Thermomicrobia (class) | Oligotroph |
| Bacteria | Cyanobacteria | Xenococcaceae (family) | Copiotroph |
| Bacteria | Deltaproteobacteria | Syntrophobacteraceae (family) | Copiotroph |
| Fungi | Ascomycota | Botryosphaeriaceae (family) | **Pathotroph** |
| Fungi | Ascomycota | Didymosphaeriaceae (family) | **Pathotroph** |
| Fungi | Ascomycota | *Ophiobolus_malleolus (species)* | **Pathotroph** |
| Fungi | Ascomycota | *Alternaria (genus)* | **Pathotroph** |
| Fungi | Ascomycota | *Didymocrea_sadasivanii (species*) | **Pathotroph** |
| Fungi | Ascomycota | Eurotiomycetes | Saprotroph |
| Fungi | Ascomycota | *Exophiala_jeanselmei (species)* | Saprotroph |
| Fungi | Ascomycota | *Verrucaria_macrostoma (species)* | Saprotroph |
| Fungi | Ascomycota | *Gibberella_tricincta (species)* | Saprotroph |
| Protist | Archaeplastida | Embryophyceae | Phototroph |
| Protist | Stramenopiles | Bacillariophyta | Phototroph |
|  |  |  |  |
|  |  | **Arid** |  |
| **Module Hub (Generalist)** |  |  |  |
| Bacteria | Alphaproteobacteria | *Balneimonas (genus)* | Oligotroph |
| **Connectors (Generalist)** |  |  |  |
| Bacteria | Alphaproteobacteria | *Pseudonocardia (genus)* | Copiotroph |

*Table S5 Pearson correlation coefficients (r) between network modules and soil variables (with the p-value in parentheses). Bold indicates significant correlations (p<0.05).*

| **Network** | **Module** | **pH** | **Total.N** | **Total.C** | **CN.ratio** | **Total.P** | **CP.ratio** | **NP.ratio** | **Sand** | **Silt** | **Clay** | **VR** | **SB** |
| --- | --- | --- | --- | --- | --- | --- | --- | --- | --- | --- | --- | --- | --- |
| **Semi-arid Low CV** | **1** | -0.15  (0.6) | 0.18  (0.5) | 0.22  (0.4) | 0 .074  (0.8) | 0.048  (0.8) | 0.18  (0.5) | 0.15  (0.5) | 0.067  (0.8) | 0.089  (0.7) | -0.16  (0.5) | 0.22  (0.4) | 0.45  (0.06) |
|  | **2** | -0.025  (0.9) | 0.18  (0.5) | 0.14  (0.6) | 0.0023  (1) | -0.055  (0.8) | 0.14  (0.6) | 0.19  (0.5) | 0.012  (1) | 0.1  (0.7) | -0.086  (0.7) | 0.17  (0.5) | **0.49**  **(0.04)** |
|  | **3** | 0.15  (0.6) | 0.14  (0.6) | -0.0011  (1) | -0.15  (0.6) | **0.6**  **(0.008)** | -0.13  (0.6) | 0.017  (0.9) | -0.34  (0.2) | 0.43  (0.08) | 0.22  (0.4) | 0.36  (0.1) | 0.14  (0.6) |
|  | **4** | 0.42  (0.08) | 0.062  (0.8) | -0.2  (0.4) | -0.27  (0.3) | 0.33  (0.2) | -0.27  (0.3) | 0.0064  (1) | -0.39  (0.1) | 0.31  (0.2) | 0.39  (0.1) | 0.38  (0.1) | 0.024  (0.9) |
|  | **5** | 0.13  (0.6) | 0.17  (0.5) | -0.014  (1) | -0.2  (0.4) | 0.21  (0.4) | -0.064  (0.8) | 0.13  (0.6) | -0.28  (0.3) | 0.3  (0.2) | 0.22  (0.4) | 0.28  (0.3) | 0.22  (0.4) |
|  | **6** | 0.044  (0.9) | -0.23  (0.4) | 0.071  (0.8) | 0.34  (0.2) | -0.41  (0.09) | 0.17  (0.5) | -0.11  (0.7) | 0.17  (0.5) | -0.16  (0.5) | -0.15  (0.6) | -0.093  (0.7) | 0.31  (0.2) |
|  | **7** | -0.36  (0.1) | -0.17  (0.5) | 0.18  (0.5) | 0.37  (0.1) | **-0.53**  **(0.02)** | 0.28  (0.3) | -0.057  (0.8) | 0.44  (0.07) | -0.45  (0.06) | -0.36  (0.1) | -0.31  (0.2) | -0.12  (0.6) |
|  |  |  |  |  |  |  |  |  |  |  |  |  |  |
| **Semi-arid High CV** | **1** | -0.21  (0.4) | -0.073  (0.8) | -0.051  (0.8) | -0.095  (0.7) | -0.44  (0.07) | -0.0015  (1) | -0.017  (0.9) | 0.16  (0.5) | 0.036  (0.9) | -0.44  (0.07) | 0.43  (0.07) | 0.21  (0.4) |
|  | **2** | -0.23  (0.3) | -0.22  (0.4) | -0.22  (0.4) | -0.27  (0.3) | -0.4  (0.1) | -0.18  (0.5) | -0.17  (0.5) | 0.095  (0.7) | 0.22  (0.4) | **-0.47**  **(0.05)** | **0.6**  **(0.009)** | -0.06  (0.8) |
|  | **3** | -0.2  (0.4) | 0.22  (0.4) | 0.19  (0.4) | 0.14  (0.6) | -0.1  (0.7) | 0.22  (0.4) | 0.24  (0.3) | 0.01  (1) | -0.14  (0.6) | -0.041  (0.9) | -0.093  (0.7) | -0.047  (0.9) |
|  | **4** | 0.017  (0.9) | 0.09  (0.7) | 0.046  (0.9) | -0.035  (0.9) | -0.28  (0.3) | 0.057  (0.8) | 0.09  (0.7) | 0.096  (0.7) | 0.0025  (1) | -0.19  (0.4) | -0.36  (0.1) | -0.064  (0.8) |
|  | **5** | 0.17  (0.5) | -0.03  (0.9) | -0.075  (0.8) | -0.06  (0.8) | 0.29  (0.3) | -0.12  (0.6) | -0.074  (0.8) | -0.15  (0.6) | 0.036  (0.9) | 0.33  (0.2) | -0.26  (0.3) | -0.22  (0.4) |
|  | **6** | -0.2  (0.4) | -0.038  (0.9) | -0.094  (0.7) | -0.085  (0.7) | 0.44  (0.07) | -0.15  (0.6) | -0.1  (0.7) | -0.38  (0.1) | 0.28  (0.3) | 0.36  (0.1) | 0.1  (0.7) | **-0.6**  **(0.009)** |
|  |  |  |  |  |  |  |  |  |  |  |  |  |  |
| **Arid** | **1** | -0.16  (0.5) | -0.28  (0.3) | -0.24  (0.3) | 0.011  (1) | 0.003  (1) | -0.25  (0.3) | -0.25  (0.3) | 0.21  (0.4) | -0.082  (0.7) | -0.21  (0.4) | **-0.54**  **(0.02)** | -0.39  (0.1) |
|  | **2** | 0.14  (0.6) | 0.0052  (1) | -0.07  (0.8) | -0.1  (0.7) | -0.03  (0.9) | -0.072  (0.8) | 0.0061  (1) | 0.13  (0.6) | 0.2  (0.4) | -0.23  (0.3) | -0.35  (0.2) | -0.17  (0.5) |
|  | **3** | **-0.59**  **(0.009)** | -0.082  (0.7) | 0.11  (0.7) | 0.46  (0.06) | -0.061  (0.8) | 0.074  (0.8) | -0.064  (0.8) | -0.02  (0.9) | -0.21  (0.4) | 0.11  (0.7) | -0.41  (0.09) | **-0.69**  **(0.002)** |
|  | **4** | -0.25  (0.3) | -0.047  (0.9) | 0.015  (1) | 0.085  (0.7) | -0.12  (0.6) | 0.0044  (1) | -0.04  (0.9) | -0.16  (0.5) | 0.026  (0.9) | 0.18  (0.5) | 0.06  (0.8) | -0.32  (0.2) |
